# The Potential Role of Vitamin D in *BRCA1* Pathogenic Variant Carriers: A Narrative Review

**DOI:** 10.3390/ijms27125545

**Published:** 2026-06-19

**Authors:** Joanna Robaczyńska, Milena Kiljańczyk, Maciej Maj, Adam Kiljańczyk, Tomasz Byrski, Cezary Cybulski, Izabela Janiuk, Jacek Gronwald, Jan Lubiński

**Affiliations:** 1 Department of Genetics and Pathology, International Hereditary Cancer Center, Pomeranian Medical University, ul. Unii Lubelskiej 1, 71-252 Szczecin, Poland; s089400@student.wum.edu.pl (J.R.); milena.matuszczak@pum.edu.pl (M.K.); cezary.cybulski@pum.edu.pl (C.C.);; 2 Department of Histology and Embryology, Medical University of Warsaw, 02-004 Warsaw, Poland; 3 Department of Diagnostic Imaging and Interventional Radiology, Pomeranian Medical University Hospital, No. 1, 71-252 Szczecin, Poland; 4 Department of Oncology and Chemotherapy, Pomeranian Medical University, 71-252 Szczecin, Poland

**Keywords:** vitamin D, vitamin D receptor, BReast CAncer gene 1, breast cancer, ovarian cancer

## Abstract

Vitamin D is a fat-soluble secosteroid essential for skeletal development and calcium homeostasis, but it also exerts pleiotropic effects on numerous biological processes via its active metabolites. Vitamin D metabolites act as steroid hormones that regulate cell-cycle progression, proliferation, differentiation, apoptosis, immune responses, and multiple intracellular signaling pathways. Moreover, they modulate the expression of genes involved in carcinogenesis. As circulating vitamin D levels are influenced by diet, fortified foods, and supplementation, they represent a potentially modifiable factor. Whether vitamin D status affects cancer risk or disease progression in carriers of pathogenic *BRCA1* variants remains unclear and continues to be actively investigated. Clarifying this relationship could have significant clinical implications for risk stratification and prevention in this high-risk population. This narrative review summarizes current evidence from epidemiological, clinical, and molecular studies examining the role of vitamin D in *BRCA1* pathogenic variant carriers. It also highlights key limitations in the existing literature and identifies critical directions for future research, emphasizing the need for well-designed prospective studies in representative cohorts.

## Simple Summary

BReast CAncer gene 1 (*BRCA1*) mutation carriers face a high lifetime risk of breast and ovarian cancer. However, modifiable environmental and lifestyle factors may influence disease onset and progression. Vitamin D is a fat-soluble secosteroid known for its role in bone metabolism, but it also regulates cell proliferation, apoptosis, immune responses and genomic stability. Emerging evidence suggests that vitamin D signaling may impact pathways that are disrupted in *BRCA1*-deficient cells. This may influence tumor development. This review summarizes current molecular and clinical research on vitamin D in *BRCA1* pathogenic variant carriers. Moreover, it highlights limitations of existing studies and identifies areas for future investigation. Understanding whether vitamin D status can modify cancer risk could inform prevention strategies and personalized care for this high-risk population.

## 1. Introduction

Germline pathogenic variants in the Breast Cancer 1 (*BRCA1*) gene confer a markedly increased lifetime risk of breast cancer (approximately a 55–72% risk of breast cancer by age 70, compared to ~12% in the general population) and ovarian cancer (around ~40%) [1]. *BRCA1* carriers more often develop early-onset disease, aggressive phenotypes (triple-negative breast cancer; TNBC), and impaired DNA damage response (DDR) [1,2,3]. Despite advances in screening, surgery, and targeted therapies, many *BRCA1* carriers still develop cancer, underscoring the importance of identifying any modifiable risk factors [1,4].

Vitamin D has been proposed as a potential candidate; however, the available evidence remains limited and inconclusive. Its biologically active form, calcitriol, exerts genomic and non-genomic effects through the vitamin D receptor (VDR), influencing cell-cycle control, apoptosis, differentiation, immune regulation, oxidative stress responses, and multiple oncogenic signaling pathways [5,6]. Importantly, vitamin D signaling also modulates the expression and activity of genes involved in DDR and genomic stability-processes that are fundamentally disrupted in *BRCA1*-deficient cells [6,7].

Circulating vitamin D levels are modifiable through diet, supplementation, and sun exposure, making vitamin D status an attractive candidate as a potential risk modifier [8]. Some epidemiological studies in the general population have suggested inverse associations between vitamin D levels and cancer incidence, tumor aggressiveness, and mortality, particularly in breast cancer, although results are inconsistent [9,10,11]. Evidence did not demonstrate a reduction in overall cancer incidence with vitamin D supplementation [12]. Nevertheless, a signal for reduced metastatic cancer rates was observed in secondary analyses [13]. It remains unclear whether these general-population findings apply to *BRCA1* carriers, whose cancers are driven by defective DNA repair. Experimental evidence suggests that vitamin D signaling intersects with pathways involved in genomic stability and DNA damage responses that are disrupted in *BRCA1*-deficient cells. However, whether these interactions translate into clinically meaningful effects in BRCA1 pathogenic variant carriers remains uncertain. In this review, we synthesize the mechanistic and clinical evidence on vitamin D and *BRCA1*-related carcinogenesis. We first describe how calcitriol regulates cell-cycle checkpoints, apoptosis, differentiation, immunity, and DNA repair processes central to *BRCA1*-deficient cells. We then review human studies of vitamin D and cancer risk in *BRCA1* carriers, noting that data are currently very limited. Finally, we highlight gaps in the literature and outline key future research directions.

## 2. Materials and Methods

This study was conducted as a narrative review aimed at summarizing current molecular, experimental, and clinical evidence regarding the relationship between vitamin D signaling and carcinogenesis in carriers of pathogenic *BRCA1* variants. A comprehensive literature search was performed using the electronic database PubMed/MEDLINE up to February 2026. The following keywords were used: vitamin D, calcitriol, vitamin D receptor, VDR, *BRCA1*, *BRCA1* mutation, *BRCA1* deficiency, breast cancer, ovarian cancer, and DNA repair. In total, 453 records were screened, of which 68 articles that best matched the scope of this review were included. Additional records were identified through reference screening of included articles (*n* = 2). Records were excluded if they were letters to the editor, editorials, research protocols, case reports, brief correspondence, non-English articles, animal studies, studies involving multiple cancer types, or studies with fewer than 20 patients. Emphasis was placed on study design quality, reproducibility of findings, sample size, and potential confounding factors [e.g., body mass index (BMI) and supplementation behavior] in epidemiological studies. An important methodological consideration is the heterogeneity of vitamin D-related exposures evaluated across the included studies. Experimental studies most investigated the biological activity of the hormonally active metabolite—calcitriol—whereas epidemiological and clinical studies generally assessed circulating 25(OH)D concentrations or vitamin D_3_ supplementation. In many reports, the exact vitamin D form was not consistently specified, making direct comparisons between studies difficult. Therefore, the present review focuses primarily on the biological and clinical effects attributed to vitamin D signaling rather than on differences between individual vitamin D compounds. This distinction should be considered when interpreting the available evidence.

## 3. Molecular Mechanisms Linking Vitamin D with BRCA1 and Carcinogenesis

### 3.1. Cell Cycle Regulation and Apoptosis

Calcitriol exerts antiproliferative and pro-apoptotic effects in breast epithelial cells through activation of the vitamin D receptor (VDR). One of its best-characterized actions is induction of G1 cell-cycle arrest through upregulation of cyclin-dependent kinase inhibitors p21 (CDKN1A) and p27 (CDKN1B), accompanied by suppression of cyclins and cyclin-dependent kinases required for G1/S transition [6]. In parallel, calcitriol promotes apoptosis through activation of caspase-3 and caspase-7 and modulation of Bcl-2 family proteins, including downregulation of anti-apoptotic Bcl-2 and Bcl-xL and upregulation of pro-apoptotic Bax and Bak [6,14,15].

Vitamin D signaling also interacts with major pathways regulating cell growth and survival. Calcitriol has been reported to inhibit the PI3K/AKT/mTOR and MAPK signaling cascades, while functional crosstalk between VDR and p53 further enhances antiproliferative and pro-apoptotic responses [14,15]. Through these mechanisms, vitamin D reduces cellular proliferation and promotes elimination of damaged cells.

These effects may be particularly relevant in *BRCA1*-associated carcinogenesis. Experimental studies suggest that calcitriol can stabilize TP53BP1 (53BP1), a key mediator of DNA damage responses, and promote p21 expression through epigenetic regulation, thereby reinforcing cell-cycle checkpoints and supporting genome maintenance in genetically unstable cells [16]. Vitamin D signaling has also been linked to epithelial differentiation and increased E-cadherin expression, features generally associated with reduced invasiveness and a less aggressive tumor phenotype [17].

Collectively, available evidence indicates that calcitriol acts as a pleiotropic regulator of breast cancer cell fate by integrating cell-cycle control, apoptosis, differentiation, and genome maintenance pathways. Although these observations originate primarily from experimental models, they provide a biologically plausible rationale for investigating whether vitamin D signaling may influence tumor development in *BRCA1*-deficient cells [16,18,19,20].

### 3.2. DNA Repair and Genome Stability

Vitamin D has been increasingly recognized as a regulator of genomic stability through its influence on DDR pathways and DNA repair mechanisms. The biologically active form of vitamin D, 1,25(OH)_2_D_3_, exerts its effects primarily via the VDR, which functions as a transcription factor controlling the expression of numerous genes involved in cell cycle regulation, oxidative stress responses, and DNA repair processes [21,22,23]. One of the best-characterized antiproliferative mechanisms of vitamin D signaling is the induction of cyclin-dependent kinase inhibitors p21(CDKN1A) and p27 (CDKN1B), resulting in inhibition of cyclin-CDK complexes, reduced phosphorylation of retinoblastoma protein (Rb), suppression of E2F transcription factor activity, and blockade of the G1-to-S phase transition [24,25].

Several studies have demonstrated that vitamin D signaling modulates the expression of key genes involved in the DDR, including *BRCA1*, Ataxia-Telangiectasia Mutated (*ATM*), and TP53. Experimental models have shown that vitamin D can upregulate *BRCA1* expression at the transcriptional level, suggesting a potential role in maintaining DNA repair capacity under physiological conditions [26,27]. Similarly, components of the ATM-dependent signaling cascade, which coordinate cellular responses to DNA double-strand breaks, may be influenced by vitamin D status, further supporting a link between vitamin D signaling and genome surveillance mechanisms [28].

Vitamin D has also been implicated in the regulation of homologous recombination (HR), a DNA repair pathway that is critically dependent on functional *BRCA1*. By influencing cell cycle checkpoint control and the expression of DNA repair genes, vitamin D may indirectly affect HR efficiency [19,26]. However, it is important to emphasize that, despite these regulatory effects, there is currently no evidence that vitamin D can functionally compensate for *BRCA1* deficiency.

In this context, *BRCA1* function should be considered within a broader homologous recombination repair network that includes key downstream mediators such as RAD51. RAD51 plays a central role in homologous recombination by facilitating strand invasion and exchange during DNA double-strand break repair, while also contributing to replication fork protection under conditions of replication stress. In *BRCA1*-deficient cells, impaired RAD51 recruitment and defective fork stabilization represent critical mechanisms underlying genomic instability. Conversely, increased RAD51 expression or restoration of RAD51 loading may partially compensate for homologous recombination defects, thereby stabilizing replication forks and promoting resistance to DNA-damaging therapies, including platinum-based chemotherapy, radiotherapy, and PARP inhibitors. These observations highlight that *BRCA1*-associated tumorigenesis and treatment response are determined not only by *BRCA1* loss itself, but also by the functional status of downstream DNA repair effectors within the broader DDR network [29].

In *BRCA1*-mutated cells, the core defect in homologous recombination persists, and vitamin D has not been shown to restore HR proficiency or fully correct the underlying DNA repair deficiency [30,31].

Beyond its direct effects on DNA repair pathways, vitamin D contributes to genome stability through its antioxidant and anti-inflammatory properties. Vitamin D signaling has been shown to reduce intracellular reactive oxygen species (ROS) levels by upregulating antioxidant enzymes and suppressing pro-oxidative signaling pathways [32]. Since oxidative stress is a major source of endogenous DNA damage, including single- and double-strand breaks, a reduced ROS burden may lower the rate of DNA lesion accumulation and decrease replicative stress [33].

In this context, vitamin D may act primarily as a genome-protective modulator rather than a direct substitute for DNA repair machinery. By limiting oxidative DNA damage, enhancing checkpoint signaling, and maintaining a cellular environment conducive to accurate DNA repair, vitamin D may help preserve genomic integrity in normal cells and delay malignant transformation. Nevertheless, in the context of inherited defects in DNA repair genes, such as pathogenic *BRCA1* variants, these protective effects—while biologically plausible—are unlikely to be sufficient to counteract the substantial repair deficits that drive genomic instability and elevate cancer risk [16,30].

### 3.3. Cellular Differentiation and EMT Inhibition

Beyond cell-cycle arrest, calcitriol exerts a well-established pro-differentiating effect, promoting epithelial lineage commitment through induction of epithelial markers and junctional components [21,34]. In breast cancer models, calcitriol stimulates the expression of differentiation-associated features, including casein production, lipid droplet formation, and adhesion proteins, reflecting a shift toward a more differentiated and less aggressive cellular phenotype [21].

At the molecular level, calcitriol suppresses β-catenin signaling by promoting its redistribution from the nucleus to adherens junctions, thereby attenuating transcriptional programs associated with proliferation and tumor progression. Concomitantly, vitamin D upregulates E-cadherin, a central epithelial adhesion molecule inversely correlated with metastatic potential, while downregulating cadherin-17, which has been linked to tumor progression. This cadherin switch reinforces epithelial integrity and may restrict metastatic dissemination [21,34].

Calcitriol further inhibits epithelial–mesenchymal transition (EMT), resulting in reduced cellular plasticity, invasiveness, and metastatic capacity. These antitumorigenic effects are mediated through coordinated modulation of major oncogenic signaling networks, including WNT/β-catenin, NF-κB, and PI3K/AKT pathways, which collectively regulate tumor progression and phenotypic plasticity.

In vivo evidence supports a critical role for vitamin D in metastasis control. Vitamin D deficiency enhances carcinogenesis, whereas intravenous calcitriol administration significantly reduces lung metastasis formation [35]. This effect is mediated by suppression of EMT and regulation of the CXCL12–CXCR4 signaling axis. Vitamin D deficiency promotes increased CXCL12–CXCR4 co-localization, facilitating metastatic dissemination [35]. These findings identify vitamin D deficiency as a potential risk factor for metastasis and suggest that adequate vitamin D status may enhance the efficacy of conventional anti-cancer therapies aimed at limiting tumor spread [35].

Additionally, calcitriol suppresses tumor invasion through regulation of extracellular matrix remodeling, including inhibition of matrix metalloproteinases (notably MMP-9) and downregulation of tenascin-C and integrins such as integrin α6 and integrin β4 [34]. Collectively, these mechanisms contribute to stabilization of the epithelial phenotype and suppression of metastatic progression.

### 3.4. Immunomodulation and Tumor Microenvironment

Vitamin D influences the tumor microenvironment through immunomodulatory and anti-inflammatory effects. Immune cells express the vitamin D receptor (VDR) and possess the enzymatic machinery required for local vitamin D metabolism, allowing vitamin D signaling to directly regulate immune responses. Experimental studies indicate that vitamin D can suppress pro-inflammatory cytokine pathways, including IL-17/IL-23 signaling, reduce IL-6 production, and promote differentiation of regulatory T cells (Tregs), thereby contributing to a more immunoregulatory microenvironment [36,37,38].

Vitamin D has also been reported to modulate macrophage function and inhibit cyclooxygenase-2 (COX-2), further limiting inflammation-associated tumor-promoting signals [38]. The relevance of these effects may be particularly important in tumors characterized by defective DNA repair. *BRCA1* deficiency and homologous recombination impairment can promote genomic instability, resulting in cytosolic DNA accumulation and activation of the cGAS–STING pathway.

This process may enhance antitumor immune responses but can also induce adaptive immune-evasion mechanisms, including increased PD-L1 expression [29]. In this context, vitamin D signaling may influence interactions between genomic instability and tumor immunity. However, the clinical significance of these mechanisms in *BRCA1* pathogenic variant carriers remains uncertain.

Overall, available evidence supports a role for vitamin D in immune regulation and inflammation control, although direct evidence linking these effects to cancer prevention or progression in *BRCA1* mutation carriers remains limited.

### 3.5. Hormonal Regulation

**Figure 1:**
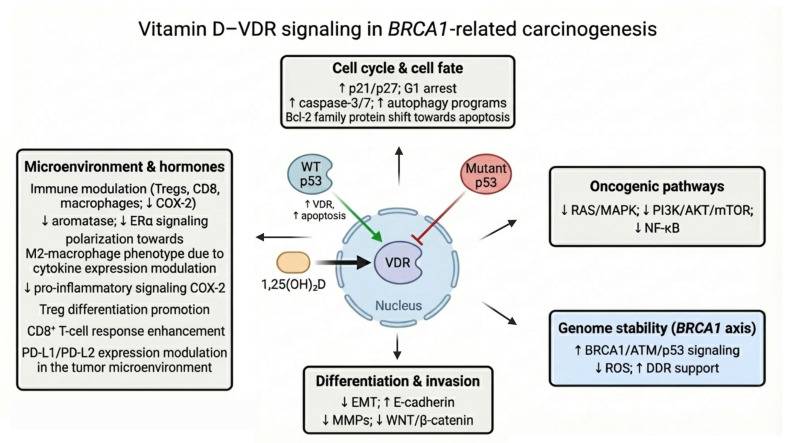
Proposed molecular mechanisms linking vitamin D–vitamin D receptor (VDR) signaling and *BRCA1*-related carcinogenesis. Created in BioRender. Maj, M. (2026) https://BioRender.com/set5r08 (accessed 13th June 2026). Active vitamin D regulates VDR-mediated pathways involved in cell-cycle control, apoptosis, differentiation, invasion, oncogenic signaling, genome stability, and tumor microenvironment modulation in *BRCA1*-associated cancers. Abbreviations: 1,25(OH)_2_D, 1,25-dihydroxyvitamin D_3_ (calcitriol); AKT, protein kinase B; ATM, ataxia telangiectasia mutated; Bcl-2, B-cell lymphoma 2; *BRCA1*, breast cancer susceptibility gene 1; CD8, cluster of differentiation 8; COX-2, cyclooxygenase-2; DDR, DNA damage response; EMT, epithelial–mesenchymal transition; ERα, estrogen receptor alpha; IL, interleukin; MAPK, mitogen-activated protein kinase; MMPs, matrix metalloproteinases; mTOR, mammalian target of rapamycin; NF-κB, nuclear factor kappa-light-chain-enhancer of activated B cells; PD-L1, programmed death-ligand 1; PD-L2, programmed death-ligand 2; PI3K, phosphatidylinositol 3-kinase; RAS, rat sarcoma proto-oncogene family; ROS, reactive oxygen species; Tregs, regulatory T cells; VDR, vitamin D receptor; WNT, Wingless/Integrated signaling pathway; WT, wild type. Green arrow indicates activation, whereas red T-shaped line indicates inhibition.

Regarding hormonal regulation, vitamin D exerts a significant modulatory effect on aromatase activity and estrogen signaling. Calcitriol suppresses aromatase transcription via promoter II, acting either directly or indirectly—through downregulation of prostaglandin E2 (PGE2), a key inducer of this promoter. Reduced aromatase activity consequently impairs local estrogen biosynthesis in breast cancer cells and surrounding breast adipose tissue, a mechanism that may be particularly relevant in *BRCA1*-associated carcinogenesis [39]. In addition, calcitriol downregulates estrogen receptor alpha (ERα) expression, further attenuating estrogen-mediated signaling. Collectively, these mechanisms diminish estrogen-driven proliferative stimuli, thereby contributing to growth suppression, especially in estrogen-dependent breast cancer [40]. The summary of all of the information mentioned above is illustrated on Figure 1.

## 4. Association Between Vitamin D Levels and Cancer Risk in BRCA1 Pathogenic Variant Carriers

Despite the strong biological rationale above, human data on vitamin D status and cancer risk in *BRCA1* carriers are sparse. No prospective cohort studies have specifically measured serum 25(OH)D in *BRCA1* mutation carriers to assess cancer incidence. The only direct evidence comes from a small case–control analysis: [41] surveyed supplement use in 134 *BRCA1/2* mutation carriers with breast cancer and 276 mutation-carrying controls. They found that women who ever used vitamin D-containing supplements had about half the odds of breast cancer compared to non-users (OR ≈ 0.54, 95% CI 0.31–0.91) [41]. Higher intake of vitamin D (often combined with calcium supplementation) was inversely associated with breast cancer risk in this high-risk group. However, these findings were derived from a small observational study and should be interpreted cautiously because causal relationships cannot be established.

For comparison, large, randomized trials in average-risk populations have not shown a benefit of vitamin D for cancer prevention. In the VITAL trial (25,871 adults randomized to 2000 IU/d vitamin D_3_ vs placebo), there was no significant difference in overall invasive cancer incidence (HR ~0.96, 95% CI 0.88–1.06) [13]. A recent meta-analysis of randomized controlled trials (RCTs) similarly concluded that vitamin D supplementation did not reduce total cancer incidence or mortality (pooled RR ≈ 1.00 for incidence, 0.93 for mortality). On the other hand, VITAL’s secondary analysis found a reduced incidence of advanced (metastatic or fatal) cancers in the vitamin D arm (HR 0.83, 95% CI 0.69–0.99) [13], an effect most evident in individuals with normal BMI. Thus, while RCTs in the general population have shown safety and possible benefit against aggressive disease, they do not support the idea that vitamin D markedly lowers overall cancer risk.

Crucially, no prospective or interventional studies have been conducted specifically in *BRCA1* carriers. The *BRCA1/2* supplement study itself notes the urgent need for cohort studies that measure baseline and on-study plasma 25(OH)D as a quantitative exposure biomarker. To our knowledge, no trial of vitamin D (or vitamin D-analog) has been performed in *BRCA1* mutation carriers. Given this evidence gap, it remains impossible to determine whether vitamin D status influences the penetrance or progression of *BRCA1*-associated cancers.

**Figure 2:**
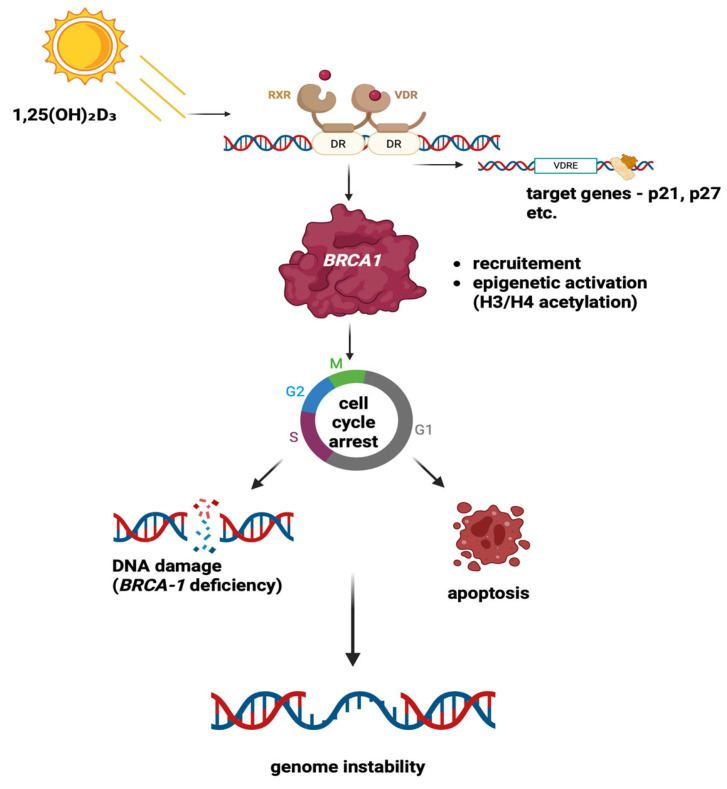
Proposed mechanistic framework linking vitamin D signaling and BRCA1-associated carcinogenesis. Created with https://BioRender.com (accessed 13 June 2026). Active vitamin D [1,25(OH)_2_D_3_] binds to the vitamin D receptor (VDR), which forms a heterodimer with the retinoid X receptor (RXR) and regulates transcription of target genes involved in cell-cycle control, including p21 and p27. BRCA1 may act as a co-regulator of VDR-dependent transcription, promoting cell-cycle arrest, apoptosis, and maintenance of genomic integrity. In contrast, *BRCA1* deficiency impairs DNA repair, leading to DNA damage accumulation and genomic instability. Together, these interactions provide a biologically plausible mechanism through which vitamin D signaling may influence BRCA1-associated cancer development. Abbreviations: 1,25(OH)_2_D_3_, 1,25-dihydroxyvitamin D_3_ (calcitriol); BRCA1, breast cancer susceptibility gene 1; DNA, deoxyribonucleic acid; H3, histone H3; H4, histone H4; RXR, retinoid X receptor; VDR, vitamin D receptor; VDRE, vitamin D response element.

Beyond cytostatic effects, vitamin D signaling may increase susceptibility of damaged cells to apoptosis and interact with pathways involved in genomic integrity [42,43]. These observations provide a biologically plausible rationale for further investigation of vitamin D in *BRCA1-*associated carcinogenesis. Nevertheless, current human evidence remains limited and insufficient to establish a causal relationship between vitamin D status and cancer risk in *BRCA1* pathogenic variant carriers. A summary of the currently available studies is presented in Table 1. The proposed mechanistic framework linking vitamin D signaling with *BRCA1*-associated carcinogenesis is summarized in Figure 2.

Importantly, the available evidence differs substantially according to study population. The only study conducted specifically in *BRCA1/2* mutation carriers reported an inverse association between vitamin D supplementation and breast cancer risk; however, the findings were derived from an observational case–control design and are susceptible to residual confounding. In contrast, randomized controlled trials and meta-analyses conducted in average-risk populations did not demonstrate a reduction in overall cancer incidence, although some evidence suggested a decrease in advanced or metastatic disease. Consequently, the current evidence base in *BRCA1* pathogenic variant carriers remains limited and should not be inferred directly from studies performed in the general population.

## 5. VDR Polymorphisms and Vitamin D Metabolism

Single-nucleotide polymorphisms (SNPs) in the VDR gene have been extensively investigated as potential modifiers of cancer susceptibility, particularly in breast cancer. The VDR encodes a nuclear hormone receptor that mediates the biological effects of 1,25(OH)_2_D_3_ by regulating transcription of genes involved in cell cycle control, apoptosis, differentiation, immune modulation, and DNA repair. Functional variation within the VDR gene may therefore influence pathways that are also disrupted in hereditary breast cancer syndromes [45,46,47].

Among the most frequently studied VDR polymorphisms are *FokI* (rs2228570), *BsmI* (rs1544410), *ApaI* (rs7975232), and *TaqI* (rs731236) [48]. Meta-analyses suggest that the *FokI* polymorphism, which alters the length and transcriptional activity of the VDR protein, is the variant most consistently associated with breast cancer risk, although effect sizes are modest and population-dependent [45,46,49,50]. Associations reported for *BsmI*, *ApaI*, and *TaqI* are less consistent, with significant heterogeneity across studies [46,47].

The potential relevance of VDR polymorphisms in carriers of *BRCA1* mutations lies in their capacity to modify cellular responses to vitamin D signaling. *BRCA1* plays a central role in DNA damage repair and maintenance of genomic stability; loss of *BRCA1* function leads to increased susceptibility to malignant transformation. It has been hypothesized that favorable vitamin D signaling, driven by functional VDR variants and adequate vitamin D availability, could partially compensate for *BRCA1* deficiency by enhancing cell cycle control and DNA repair pathways. Conversely, unfavorable *VDR* genotypes or impaired vitamin D metabolism could exacerbate genomic instability and tumorigenesis [16].

Empirical evidence directly addressing gene-gene interactions between *BRCA1* and *VDR* remains limited. A small number of case–control studies have examined *VDR* polymorphisms in breast cancer patients with known *BRCA1/2* germline mutation status, suggesting possible associations but lacking sufficient power to draw definitive conclusions [51]. Other studies have reported associations between VDR polymorphisms and breast cancer risk in *BRCA1/2*-negative populations, supporting the concept of VDR as a low-penetrance modifier rather than a primary susceptibility gene [52].

Beyond VDR itself, genetic variation in other components of the vitamin D pathway, including genes involved in vitamin D synthesis, transport, and catabolism, has been investigated in relation to breast cancer risk. These studies further support the notion that interindividual variability in vitamin D metabolism may influence cancer susceptibility, potentially through interactions with environmental factors such as dietary vitamin D and calcium intake [50,53].

Overall, while the hypothesis that vitamin D metabolism and VDR polymorphisms modulate *BRCA1* mutation penetrance is biologically plausible, current evidence remains largely indirect and inconclusive. The genetic interaction between *BRCA1* and VDR is insufficiently explored, and existing studies are limited by small sample sizes, population heterogeneity, and lack of functional validation. Nonetheless, this represents a promising area for future research, with potential implications for risk stratification and personalized prevention strategies in *BRCA1* mutation carriers [16,45,51]. Key modifiers that may affect vitamin D activity and interpretation of existing studies are summarized in Table 2.

## 6. Should Vitamin D Be Supplemented?

Whether vitamin D supplementation confers meaningful clinical benefit remains controversial, largely due to limited and inconsistent evidence from randomized controlled trials. A large nationwide randomized study involving 25,871 participants found no significant reduction in the incidence of invasive cancer with vitamin D supplementation [44]. Notably, however, secondary analyses suggested a lower risk of metastatic disease and a modest reduction in cancer-related mortality among individuals receiving vitamin D. The strongest protective association was observed in participants with normal BMI, raising the possibility that obesity may attenuate the biological effects of vitamin D [13].

Existing reviews and translational studies indicate that vitamin D supplementation is generally safe, inexpensive, and modifiable, supporting its potential clinical relevance. Collectively, current evidence suggests that vitamin D may exert chemopreventive and prognostic effects. However, causality has not been established [12,54]. Importantly, no randomized controlled trials have specifically evaluated vitamin D supplementation in carriers of *BRCA1* pathogenic variants. Moreover, optimal serum vitamin D concentration, timing of supplementation (preventive versus post-diagnosis), and its potential influence on mutation penetrance remain undefined.

No prospective cohort or intervention study has specifically measured circulating 25(OH)D levels in *BRCA1* carriers to test their effect on cancer risk or outcomes [55]. In fact, the authors of the only available *BRCA1/2* supplement study explicitly highlight the need for prospective analyses with plasma vitamin D measurements as biomarkers of total exposure.

In summary, despite a strong biological rationale (vitamin D influences DNA repair, cell cycle, apoptosis, and immunity), human data in *BRCA1* mutation carriers are too limited to draw firm conclusions. There is currently no direct evidence that vitamin D status or supplementation alters *BRCA1* mutation penetrance or disease progression. Until well-designed prospective studies and trials are conducted, routine vitamin D supplementation beyond general health recommendations cannot be justified solely for cancer prevention in this high-risk group. Future research should focus on longitudinal studies and clinical trials that measure serum 25(OH)D (and account for genetic factors such as *VDR* variants) to determine whether maintaining adequate vitamin D status can meaningfully affect cancer risk or prognosis in *BRCA1* mutation carriers [41]. Importantly, current evidence does not support the use of vitamin D supplementation specifically for cancer prevention in BRCA1 pathogenic variant carriers beyond established recommendations for maintaining adequate vitamin D status in the general population.

## 7. Limitations of Vitamin D as a Therapeutic Agent

Although vitamin D signaling has been linked to several pathways potentially relevant for *BRCA1*-associated carcinogenesis, translating these mechanistic observations into preventive or therapeutic strategies remains challenging. Several pharmacological and biological characteristics of vitamin D limit its suitability as a conventional drug-like molecule.

Vitamin D is a highly lipophilic secosteroid with limited aqueous solubility, which strongly influences its pharmacokinetic behavior [56]. After absorption, vitamin D circulates largely bound to vitamin D-binding protein and is extensively distributed into adipose tissue and other lipid-rich compartments [56,57]. This storage capacity contributes to its relatively long half-life but also leads to considerable interindividual variability in circulating 25-hydroxyvitamin D levels [57]. As a result, the relationship between administered dose and systemic exposure is not linear and may differ substantially between individuals.

Vitamin D status is additionally influenced by numerous environmental and physiological factors, including diet, intestinal absorption, ultraviolet B exposure, body mass index, age, and genetic variability in vitamin D-metabolizing enzymes [58,59]. Obesity has been consistently associated with lower circulating vitamin D levels, partly due to volumetric dilution and increased deposition in adipose tissue [57,59]. Such variability complicates the definition of standardized supplementation regimens, particularly when considering high-risk populations such as carriers of *BRCA1* pathogenic variants.

Another limitation of the current literature is the inconsistent reporting of vitamin D forms across studies. While mechanistic studies frequently employ calcitriol, clinical investigations often evaluate vitamin D_3_ supplementation or serum 25(OH)D levels. Consequently, it remains difficult to attribute the observed biological effects to a specific vitamin D metabolite, and the available evidence should be interpreted as reflecting vitamin D pathway activity rather than the action of a single defined compound. A limitation of the present review is that it does not systematically compare vitamin D formulations, concentrations, treatment durations, or other experimental parameters across studies. Considerable heterogeneity exists among the available reports with respect to dosing regimens and study design, which may contribute to differences in the observed biological responses. Consequently, the findings summarized herein should be interpreted primarily as an overview of the reported biological effects of vitamin D rather than as evidence supporting specific treatment protocols or dosing strategies.

Another challenge relates to the complex metabolic activation of vitamin D. The hormonally active metabolite, 1,25(OH)_2_D_3_, is generated through sequential hydroxylation in the liver and kidney, mainly via CYP2R1 and CYP27B1, while CYP24A1 mediates degradation of active metabolites [56,60]. Consequently, the biological activity of vitamin D depends not only on intake or supplementation but also on hepatic and renal function as well as tissue-specific regulation of activating and inactivating enzymes.

Safety considerations represent another important limitation. High vitamin D exposure may lead to hypercalcemia, hypercalciuria, nephrolithiasis, and vascular calcification [61,62]. These effects constrain the possibility of dose escalation that might otherwise be required to reproduce the antiproliferative effects observed in experimental cancer models.

Vitamin D signaling is also intrinsically pleiotropic. Through activation of the VDR, calcitriol regulates the expression of hundreds of genes involved in calcium homeostasis, immune regulation, cellular proliferation, differentiation, and apoptosis [56,63]. While this broad transcriptional activity may underlie some of the proposed anti-cancer effects of vitamin D, it simultaneously reduces pathway specificity and increases the likelihood of systemic effects when supraphysiological doses are used.

Drug interactions may further complicate clinical use. Several commonly prescribed medications—including glucocorticoids, anticonvulsants, rifampicin, and antiretroviral agents—can enhance vitamin D catabolism through induction of cytochrome P450 enzymes [58]. Conversely, high-dose vitamin D supplementation may increase the risk of hypercalcemia in patients treated with thiazide diuretics or other drugs affecting calcium balance [62].

Importantly, evidence from large randomized clinical trials does not consistently support a protective effect of vitamin D supplementation on overall cancer incidence. In the VITAL trial, vitamin D supplementation did not significantly reduce total cancer incidence in the general population, although modest effects on cancer mortality were suggested in some analyses [44].

In the context of hereditary breast and ovarian cancer, these limitations are particularly relevant. While vitamin D may influence biological processes associated with genomic stability, inflammation, and tumor suppression, the clinical relevance of these effects in *BRCA1* mutation carriers remains uncertain. Further studies specifically addressing genetically defined high-risk populations are needed; however, current evidence remains insufficient to support a role for vitamin D supplementation in cancer risk reduction among *BRCA1* mutation carriers. Current evidence remains limited due to the small number of studies conducted specifically in *BRCA1* pathogenic variant carriers and the potential influence of confounding factors. For instance, observational case–control studies cannot establish whether vitamin D itself exerts a protective effect or merely serves as a surrogate marker of other health-promoting behaviors. Moreover, the distinct clinicopathological characteristics of breast cancer in *BRCA1* carriers, including earlier age at onset and a higher prevalence of triple-negative breast cancer, may restrict the direct extrapolation of findings from studies performed in average-risk populations. Consequently, further large-scale prospective studies are required to clarify the role of vitamin D in cancer prevention and management among *BRCA1* pathogenic variant carriers.

The take-home message is that there is currently no clinical evidence that vitamin D supplementation reduces cancer risk or modifies *BRCA1* mutation penetrance. The significance of this review lies in summarizing the molecular rationale and identifying specific gaps that should be addressed in future prospective studies.

**Figure 3:**
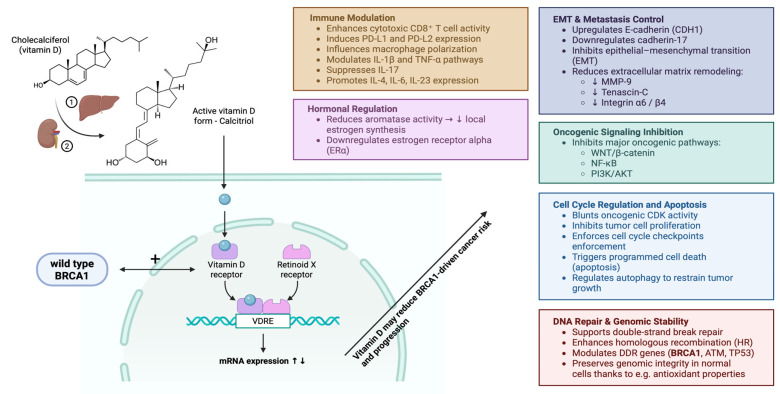
An integrated overview of the proposed relationship between vitamin D and *BRCA1*-associated carcinogenesis. Created with https://BioRender.com. (accessed 13 June 2026). Following hepatic and renal activation, vitamin D is converted to its biologically active form, calcitriol, which binds to the vitamin D receptor (VDR). The VDR forms a heterodimer with the retinoid X receptor (RXR) and interacts with vitamin D response elements (VDREs) to regulate gene transcription. Through these genomic actions, vitamin D influences multiple biological processes relevant to *BRCA1*-associated cancer development, including immune modulation, hormonal regulation, epithelial–mesenchymal transition (EMT) and metastasis control, inhibition of oncogenic signaling pathways, cell-cycle regulation and apoptosis, and maintenance of DNA repair and genome stability. Collectively, these mechanisms may modulate BRCA1-driven cancer risk and disease progression. Abbreviations: *BRCA1*, breast cancer susceptibility gene 1; CD8+, cluster of differentiation 8-positive T lymphocytes; CDH1, cadherin 1 (E-cadherin); CDK, cyclin-dependent kinase; DDR, DNA damage response; EMT, epithelial–mesenchymal transition; ERα, estrogen receptor alpha; HR, homologous recombination; IL, interleukin; MMP-9, matrix metalloproteinase-9; NF-κB, nuclear factor kappa-light-chain-enhancer of activated B cells; PI3K, phosphatidylinositol 3-kinase; RXR, retinoid X receptor; TNF-α, tumor necrosis factor alpha; TP53, tumor protein p53; VDR, vitamin D receptor; VDRE, vitamin D response element; WNT, Wingless/Integrated signaling pathway.

Figure 3 summarizes the proposed biological mechanisms underlying the protective effects of vitamin D, together with the key limitations that currently hinder translation into clinical practice.

## Figures and Tables

**Table 1:** Summary of clinical and epidemiological studies evaluating vitamin D exposure and cancer outcomes according to study population. Only one identified study specifically investigated *BRCA1/2* mutation carriers, whereas the remaining evidence derives from average-risk populations. Therefore, findings from general-population studies should not be directly extrapolated to *BRCA1* pathogenic variant carriers.

Studies in *BRCA1/2* Mutation Carriers					
Study	Study Design	Population	Vitamin D Exposure Assessment	Main Findings	Limitations
[41]	Case–control	134 *BRCA1/2* mutation carriers with breast cancer; 276 mutation carriers without cancer	Self-reported use of vitamin D and calcium supplements	Vitamin D supplement use associated with lower odds of breast cancer (OR ≈ 0.54, 95% CI 0.31–0.91)	Self-reported supplementation, potential lifestyle confounding, no serum 25(OH)D measurements
**Studies in the general population**					
**Study**	**Study Design**	**Population**	**Vitamin D Exposure Assessment**	**Main Findings**	**Limitations**
[44]	Randomized controlled trial	25,871 adults from the general population	Vitamin D_3_ supplementation (2000 IU/day)	No significant reduction in overall invasive cancer incidence	Not specific to BRCA1 mutation carriers
[13]	Secondary analysis of VITAL trial	General population	Vitamin D_3_ supplementation	Reduced incidence of advanced/metastatic cancer (HR 0.83, 95% CI 0.69–0.99)	Secondary analysis; population not genetically high-risk
[12]	Meta-analysis of randomized controlled trials	General population	Vitamin D_3_ supplementation	No significant reduction in overall cancer incidence or mortality	Heterogeneous study populations and vitamin D dosing

**Table 2:** Factors modifying vitamin D signaling and cancer risk in BRCA1 mutation carriers.

Factor	Biological Mechanism	Potential Interaction with Vitamin D/VDR Signaling	Evidence Type	Clinical Relevance
*BRCA1* variant type (truncating vs. missense)	Different degrees of homologous recombination impairment	Altered genomic stability may modify responsiveness to vitamin D-mediated DNA repair pathways	Genetic and mechanistic studies	May influence penetrance and tumor phenotype
TP53 mutation status	p53 regulates cell cycle arrest and DNA repair	Mutant p53 can interfere with VDR transcriptional activity and downstream gene regulation	Experimental and molecular studies	Could affect response to vitamin D signaling in tumors
Tumor subtype (e.g., triple-negative breast cancer)	Distinct molecular drivers and immune microenvironment	Vitamin D signaling may influence proliferation, EMT, and immune modulation differently across subtypes	Observational and mechanistic studies	Important for risk stratification and therapy response
Body mass index (BMI)	Adipose tissue sequestration of vitamin D	Lower circulating 25(OH)D levels in individuals with higher BMI	Epidemiological studies	May influence vitamin D bioavailability and supplementation needs
Vitamin D supplementation and sunlight exposure	Determines circulating 25(OH)D concentrations	Higher vitamin D levels may enhance VDR-mediated transcription and tumor-suppressive effects	Clinical and observational studies	Important confounding factors in epidemiological analyses
Hormonal environment (e.g., estrogen signaling)	Crosstalk between ER signaling and VDR pathways	Vitamin D may modulate estrogen receptor signaling and breast cancer proliferation	Experimental studies	Potential implications for hormone-responsive tumors
Therapeutic context (chemotherapy, PARP inhibitors)	DNA damage-based therapies rely on impaired repair pathways	Vitamin D may modulate DNA repair capacity and treatment sensitivity	Preclinical studies	Potential impact on therapy response

## Data Availability

No new data were created or analyzed in this study. Data sharing is not applicable to this article.

## Abbreviations

The following abbreviations are used in this manuscript:

BCa	Breast cancer
BMI	Body mass index
CI	Confidence interval
DDR	DNA damage response
DNA	Deoxyribonucleic acid
EMT	Epithelial–mesenchymal transition
ERα	Estrogen receptor alpha
G1/S	G1 to S phase transition
HR	Homologous recombination
HR (statistical)	Hazard ratio
OR	Odds ratio
RCT	Randomized controlled trial
ROS	Reactive oxygen species
SNP	Single nucleotide polymorphism
TNBC	Triple-negative breast cancer
VDR	Vitamin D receptor
